# Supplementary figures and images for: Serum miR-379 expression is related to the development and progression of hypercholesterolemia in non-alcoholic fatty liver disease
Source: PLoS One. 2020 Feb 27;15(2):e0219412. doi: 10.1371/journal.pone.0219412 (PMC7046274; doi:10.1371/journal.pone.0219412)

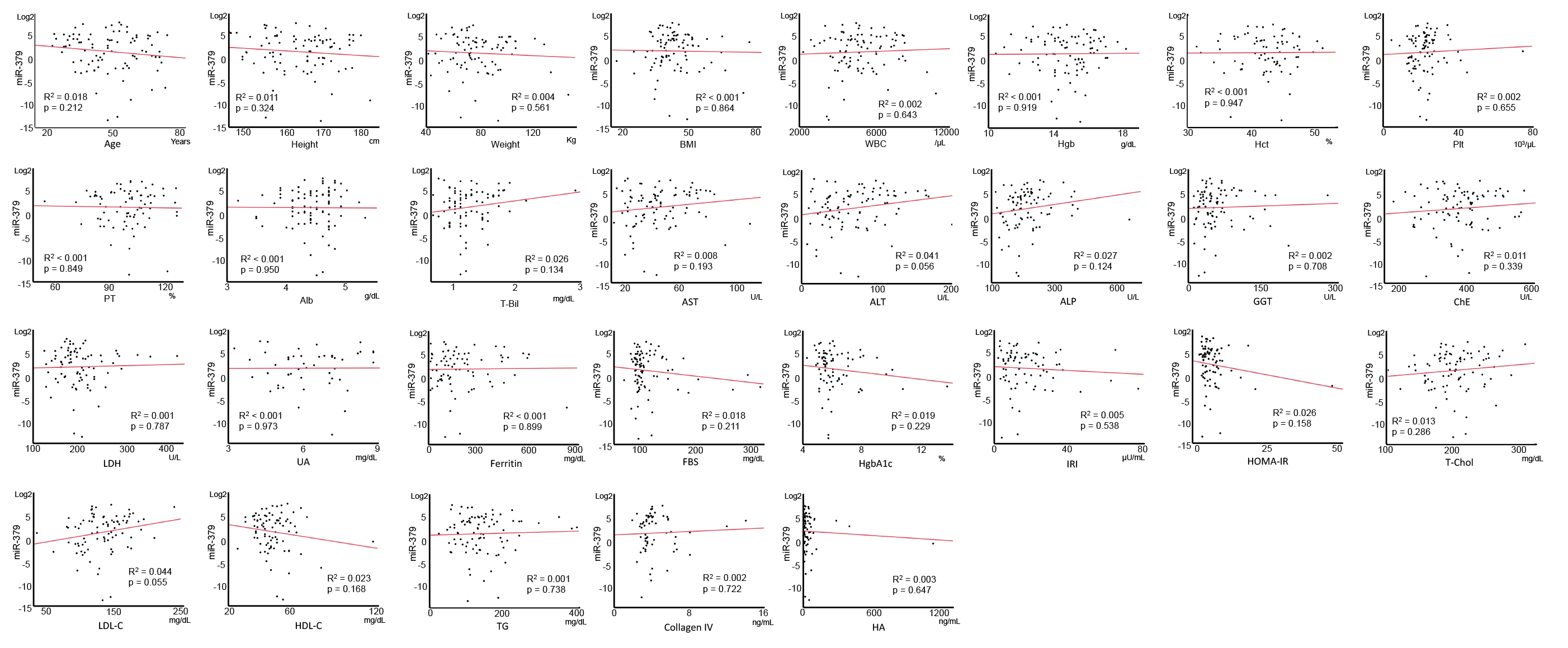

Supplement: S1 Fig — Normalized relative to serum miR-16; miR-379 values represent fold-difference relative to the normal control. (TIF) [file pone.0219412.s001.tif]

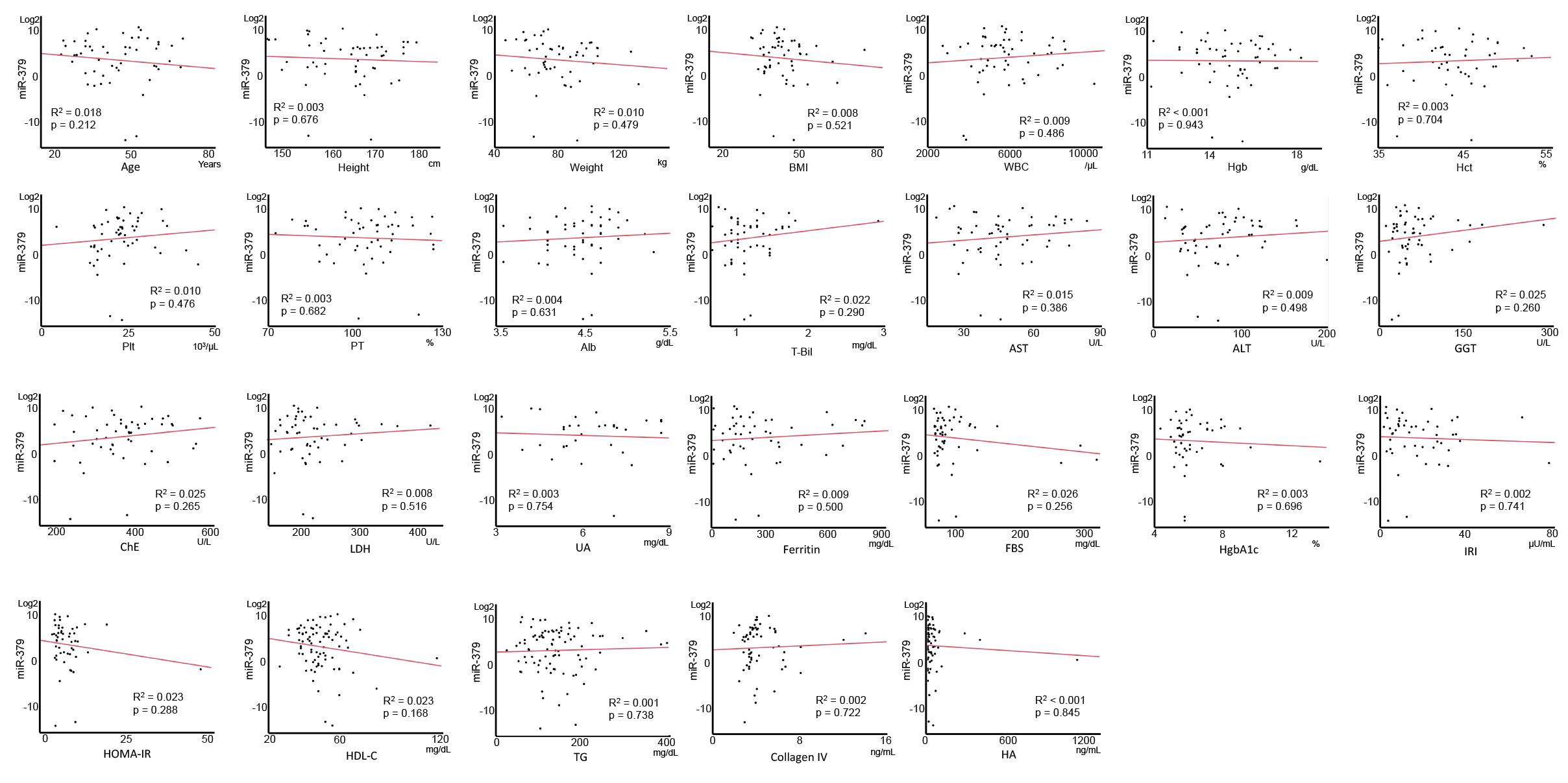

Supplement: S2 Fig — Normalized relative to serum miR-16; miR-379 values represent fold-difference relative to the normal control. (TIF) [file pone.0219412.s002.tif]

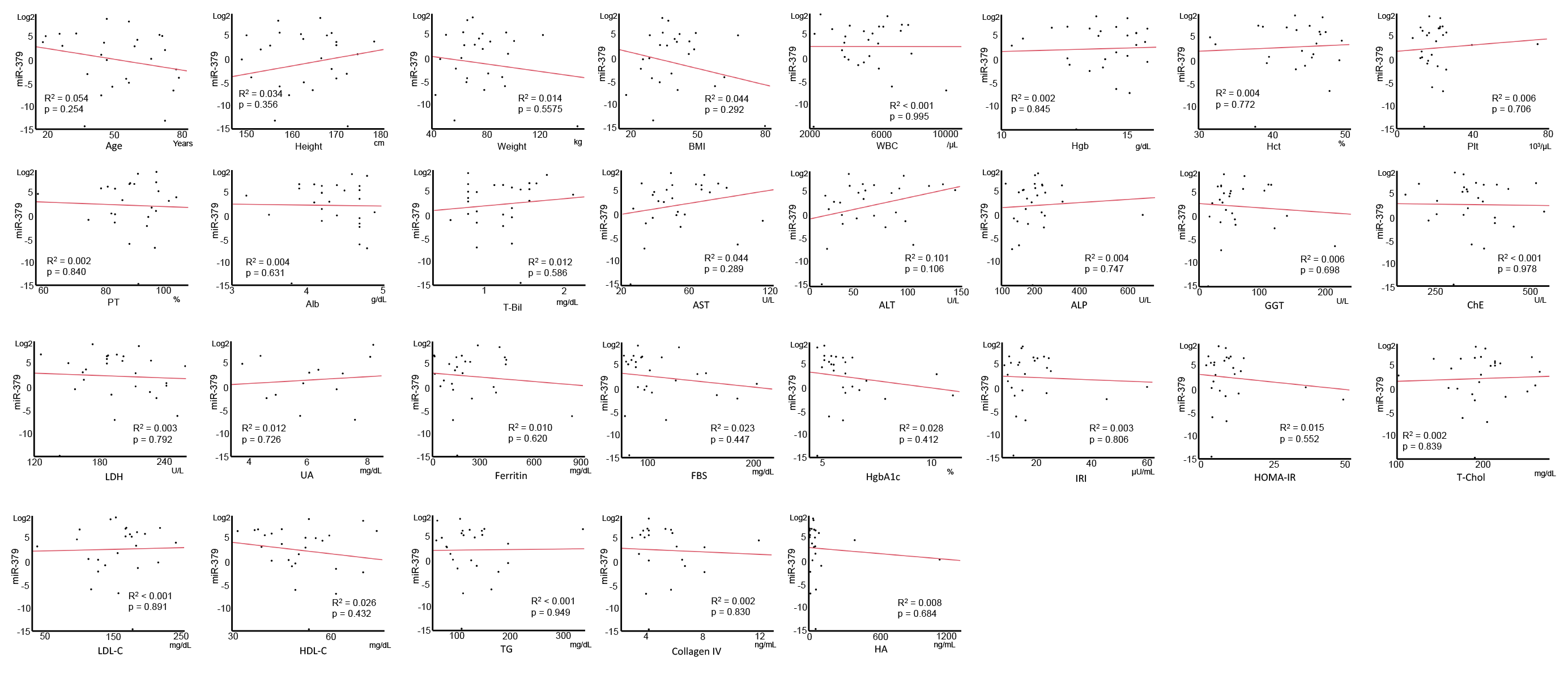

Supplement: S3 Fig — Normalized relative to serum miR-16; miR-379 values represent fold-difference relative to the normal control. (TIF) [file pone.0219412.s003.tif]
